# Urine volatile organic compounds profiling via GC-IMS combined with machine learning: a powerful diagnostic and pathogen differentiation tool for urinary tract infections

**DOI:** 10.3389/fcimb.2026.1745468

**Published:** 2026-02-11

**Authors:** Xin Zheng, Xiaohang Sun, Wenjing Du, Shoulin Sun, Dongge Chen, Wen Cheng, Xuewei Zhuang, Yanli Zhang

**Affiliations:** 1 Department of Clinical Laboratory, Shandong Provincial Third Hospital, Shandong University, Jinan, Shandong, China; 2 Department of Clinical Laboratory, Jiyang People’s Hospital of Jinan, Jinan, Shandong, China

**Keywords:** biomarker, diagnostic model, gas chromatography-ion mobility spectrometry, machine learning, precision medicine, urinary tract infection, volatile organic compounds

## Abstract

**Background:**

The diagnostic delay associated with standard urine culture necessitates rapid, accurate alternatives for urinary tract infection (UTI) management. Volatile organic compounds (VOCs) emitted by microbes represent a promising source of metabolic biomarkers for infection diagnosis.

**Objective:**

To develop and validate a diagnostic model for UTI by integrating urine VOCs profiles obtained via gas chromatography-ion mobility spectrometry (GC-IMS) with clinical features using machine learning.

**Methods:**

We conducted a prospective cohort study of 258 adults with suspected UTI. Clean-catch midstream urine samples were collected for clinical urinalysis, culture (reference standard), and GC-IMS-based VOCs analysis. VOCs and clinical data were used to train and test machine learning models (Logistic Regression, Random Forest, Support Vector Machine). Model performance was assessed by area under the receiver operating characteristic curve (AUC), sensitivity, specificity, and decision curve analysis.

**Results:**

Among 258 enrolled patients, 152 (58.9%) were culture-positive. We identified 11 differentially expressed VOCs between infected and non-infected groups, with acetic acid, benzaldehyde, and furan being the most significant (Bonferroni-adjusted *p* < 0.05). A Random Forest model integrating both VOCs and clinical features demonstrated superior performance (AUC of 0.914, with an accuracy of 82.1% (95% CI: 71.8-89.8%), sensitivity of 87.0%, specificity of 75.0%, and an F1-score of 0.851) compared to models using clinical-only (AUC 0.831) or VOC-only (AUC 0.850). Multivariate analysis confirmed acetic acid (OR 3.27) and benzaldehyde (OR 4.95) as strong independent predictors of UTI. Furthermore, VOCs profiles allowed moderate discrimination between Gram-positive and Gram-negative bacterial infections (AUC 0.800) and exhibited pathogen-specific patterns.

**Conclusion:**

The integration of urine VOCs profiles obtained by GC-IMS with routine clinical parameters using machine learning achieves high diagnostic accuracy for UTI and shows potential for rapid pathogen differentiation. This strategy could improve UTI diagnostics, enabling faster, more precise antibiotic therapy.

## Introduction

Urinary tract infection (UTI) is among the most common and burdensome bacterial infections globally, affecting hundreds of millions annually and causing significant morbidity, mortality, and substantial healthcare costs (Foxman, 2010; Zi et al., 2024). Currently, microbial identification and antimicrobial susceptibility testing based on urine culture are widely regarded as the diagnostic gold standard. However, this method has a critical weakness—a prolonged turnaround time, typically 24–48 hours (Goebel et al., 2021; Chang et al., 2025). This diagnostic delay severely hinders the timely initiation of targeted antimicrobial therapy, compelling clinicians to rely heavily on empirical, broad-spectrum antibiotics in the absence of etiological evidence. This practice significantly contributes to the escalating global crisis of antimicrobial resistance, creating a vicious cycle that urgently needs to be broken (Dunne et al., 2022; Wagenlehner et al., 2022; Onyango et al., 2024).

To bridge this diagnostic gap, point-of-care biomarkers such as urine leukocyte esterase, nitrite, and microscopic white blood cell (WBC) and bacterial count (BACT) are widely used for rapid screening. However, numerous studies indicate that the diagnostic performance of these indicators is suboptimal, with sensitivity and specificity varying considerably across different patient populations, often leading to high rates of false negatives and false positives, resulting in misdiagnosis or missed diagnoses (Gu et al., 2020; Advani et al., 2024; Kristensen et al., 2025). Consequently, developing novel UTI diagnostic tools that are rapid (within hours), accurate, and capable of providing preliminary pathogen-level information has become a shared focus and urgent need in clinical microbiology, laboratory medicine, and infectious diseases (Bermudez et al., 2025).

In recent years, volatile organic compounds (VOCs) profiles released by microorganisms during growth and metabolism have emerged as a highly promising “metabolic fingerprint,” showing great potential for the rapid diagnosis and differential diagnosis of infectious diseases (Sethi et al., 2013; Kunze-Szikszay et al., 2021). Different pathogens possess unique metabolic networks and enzyme systems; when utilizing host nutrients for proliferation, they release specific types and concentrations of VOCs, forming a distinct “odor profile” that reflects the pathogen species, abundance, and host-pathogen interactions (Bos et al., 2013; Belizario et al., 2020; Ratiu et al., 2020). This diagnostic strategy based on volatile metabolites opens new avenues for non-invasive, rapid etiological identification. Among various VOCs analysis techniques, gas chromatography-ion mobility spectrometry (GC-IMS) is particularly suitable for high-throughput VOCs fingerprinting of clinical samples due to its high sensitivity, rapid analysis, and relative operational simplicity. Its successful application in emerging infectious diseases, respiratory infections, and sepsis underscores its significant potential in medical diagnostics (Drees et al., 2019; Nazareth et al., 2022; Zhao et al., 2025).

Although pioneering studies have preliminarily confirmed the potential of urine VOCs to distinguish UTI patients from healthy controls, the current research paradigm in this field faces significant limitations. First, most studies investigate VOCs profiles in isolation, failing to integrate them deeply with routine clinical indicators, thus preventing an accurate assessment of their incremental diagnostic value within existing workflows. Second, the powerful potential of VOCs for pathogen typing (e.g., discriminating Gram stain status and even specific species) remains underexplored, leaving the rich etiological information they contain largely undecoded. Finally, from a data analysis perspective, there is a widespread failure to fully leverage advanced machine learning algorithms to extract stable diagnostic features from high-dimensional, complex VOCs data, limiting model generalizability and clinical applicability (Guernion et al., 2001; Dospinescu et al., 2020).

Therefore, several key scientific questions remain to be answered through well-designed prospective studies: First, are there stable and reproducible differential patterns in urine VOCs profiles between patients classified as infected or non-infected based on the reference standard of urine culture? Second, can integrating high-dimensional VOCs information with routine clinical parameters construct a UTI diagnostic model with performance surpassing existing standards? Finally, can VOCs-based metabolic features reliably differentiate the Gram stain characteristics and specific types of causative pathogens, thereby providing clinicians with proactive, precise therapeutic guidance before traditional susceptibility results are available? This study aims to systematically address these questions through a prospective diagnostic accuracy study, ultimately promoting a shift towards faster, more precise, and more sustainable UTI diagnosis and management.

## Methods

### Study design and participants

This was a single-center prospective diagnostic accuracy study conducted at Shandong Provincial Third Hospital from June 2024 to December 2024. The sample size was calculated based on an expected AUC of 0.85, a significance level (α) of 0.05, and a power (1-β) of 90%, yielding a minimum requirement of 200 participants. Ultimately, 258 participants were enrolled to ensure sufficient statistical power and account for potential exclusions.

Inclusion criteria were:

1. age ≥ 18 years;
2. clinical suspicion of UTI (presence of at least two of the following symptoms: frequency, urgency, dysuria, suprapubic pain, or fever >38 °C);
3. ability to provide a clean-catch midstream urine sample;
4. provision of written informed consent.

Exclusion criteria were:

1. pregnancy or lactation;
2. severe immunocompromised status (e.g., HIV infection, post-organ transplantation, long-term immunosuppressive therapy);
3. inability to comply with the study procedures.

Note: Patients with comorbidities such as diabetes mellitus were not excluded, as these conditions are common in the population with suspected UTI. Instead, these variables were recorded and included as covariates in statistical analyses to control for their potential confounding effects on urinary VOCs profiles.

### Sample collection, processing, and group definition

All participants provided clean-catch midstream urine samples. Samples were processed within 30 minutes of collection and aliquoted into three parts: one was immediately sent to the clinical laboratory for standard urinalysis with sediment analysis; another was used for standard urine culture; and a third 5 mL aliquot was transferred to a sterile container and immediately frozen at -80 °C for subsequent VOCs analysis. All samples were stored at -80 °C and analyzed within 3 months of collection, with no freeze-thaw cycles (McFarlanE et al., 2020).

Urinalysis with sediment examination was performed using a UF-5000 automated urine particle analyzer (Sysmex, Japan). Urine culture was performed using standard blood agar and MacConkey agar plates, incubated at 35 °C for 24–48 hours. Significant bacteriuria was defined as a colony count ≥ 10^5^ CFU/mL of a single uropathogen.

Based on the reference standard of urine culture, participants were classified into two groups:

(1) Infected (UTI) Group: Patients whose urine culture yielded significant growth of a single uropathogen (≥ 10^5^ CFU/mL).

Non-infected Control Group: Patients whose urine culture showed no significant growth (or growth below the diagnostic threshold)(2)

Pathogen identification was performed using the Zhongyuan Mass Spectrometry Microbial Identification System (China) and/or the VITEK 2 Compact automated system (bioMérieux, France). Antimicrobial susceptibility testing was conducted following the Clinical and Laboratory Standards Institute guidelines (CLSI M100, 34th edition).

### VOCs analysis and detection

The analysis of VOCs was conducted using gas chromatography-ion mobility spectrometry (GC-IMS; FlavorSpec, G.A.S., Dortmund, Germany). This technique separates complex VOCs mixtures first by gas chromatography (GC) employing a highly polar MXT-WAX column, and then by ion mobility spectrometry (IMS) based on the ions’ mass and collision cross-section. This two-dimensional separation yields characteristic retention indices and drift times, enabling both qualitative identification and quantitative analysis based on signal intensity. For headspace analysis, 2 mL of urine sample was incubated at 100 °C for 5 minutes, following which a 1000 μL aliquot of the headspace gas was automatically injected. High-purity nitrogen served as both the carrier and drift gas. The carrier gas flow was programmed with a gradient, while the drift gas flow was maintained at a constant 150 mL/min. Key operational temperatures were set as follows: drift tube at 45 °C, and the GC column, inlet, and transfer lines at 80 °C. Qualitative identification of VOCs was performed by matching the obtained retention indices against the NIST 2020 RI database and the relative drift times against the Hanon 2025 Dt database.

### Clinical data collection

The following data were collected from the electronic medical record system: demographic characteristics (age and sex); laboratory parameters, including blood-based markers (procalcitonin [PCT], C-reactive protein [CRP], and white blood cell [WBC] count) and urinalysis results (urinary WBC, red blood cell [RBC], and BACT, as well as nitrite status); comorbidities (diabetes mellitus and hypertension); and microbiological findings (pathogen type and Gram stain characteristic). Comorbidities such as diabetes and hypertension were specifically recorded because they may influence host metabolism and potentially alter urinary VOCs profiles. These variables were included as covariates in subsequent multivariate analyses to control for their potential confounding effects.

### Statistical analysis

All statistical analyses were conducted using Python 3.8 with the scikit-learn, statsmodels, and pandas packages. Continuous variables are presented as median (interquartile range) due to non-normal distributions, which were confirmed by Shapiro-Wilk tests (all *p* < 0.05), while categorical variables are presented as numbers and percentages. Group comparisons were performed using the Mann-Whitney U test for continuous variables and the Chi-square test for categorical variables, with a two-sided *p*-value < 0.05 indicating statistical significance. Effect sizes were calculated as η², and the Bonferroni method was applied to correct for multiple comparisons.

### Missing data handling

The extent of missing data varied by variable, reflecting real-world clinical practice where tests are selectively ordered based on disease severity. Among key infection markers: procalcitonin (PCT) and C-reactive protein (CRP) were each missing in 104 of 258 patients (40.3%); white blood cell count (WBC count) was missing in 106 patients (41.1%); red blood cell count (RBC count) was missing in 105 patients (40.7%); and bacterial count (BACT) was missing in 107 patients (41.5%). Other clinical variables had minimal missingness (<8%), and volatile organic compounds (VOCs) data were complete for all participants. Missing values were imputed with the median for continuous variables and the mode for categorical variables to enable complete-case analysis for model development. Volatile organic compounds (VOCs) data were normalized via logarithmic transformation (log1p) followed by Z-score standardization.

### Feature sets for diagnostic modeling

Three distinct feature sets were constructed and compared:

(1) Clinical-only: age, body temperature (Fever), procalcitonin (PCT), C-reactive protein (CRP), white blood cell count (WBC count), red blood cell count (RBC count), bacterial count (BACT), urinary nitrite positivity, diabetes mellitus, and hypertension.

(2) VOC-only: All 33 volatile organic compounds detected by GC-IMS (from Propanoic acid to Propanal).

Combined (Clinical+VOC): The union of clinical and VOCs features.(3)

Machine Learning Algorithms

We trained and compared three machine learning algorithms: L2-regularized logistic regression (LR), Random Forest (RF) with 100 trees, and Support Vector Machine (SVM) with a radial basis function (RBF) kernel.

Feature Selection Strategy

For diagnostic model development, we used all available VOCs features without univariate pre-filtering, as such filtering can lead to overfitting and loss of potentially informative features. However, for multivariate logistic regression analysis, we employed LASSO (least absolute shrinkage and selection operator) regularization for robust feature selection within the training data only.

Data Splitting and Model Comparability

To ensure fair comparison across models with different feature sets, we employed identical training and test splits for all models. The dataset was first split into a training set (70%) and an independent test set (30%), stratified by infection status. This same split was then applied to all feature configurations (clinical-only, VOC-only, and combined), ensuring that model comparisons were based on identical patient samples.

### Model evaluation

Model performance was evaluated using 5-fold stratified cross-validation on the training set, with the final assessment conducted on the test set. Performance metrics included the area under the receiver operating characteristic curve (AUC), accuracy, sensitivity, specificity, F1-score, and precision-recall curves. Model calibration was assessed with calibration curves, and clinical utility was quantified via decision curve analysis (DCA). The statistical significance of the model’s accuracy relative to the non-information rate (NIR) was assessed using a binomial test.

### Multivariate analysis

A multivariate logistic regression model was fitted to identify independent predictors, reported as odds ratios (ORs) with 95% confidence intervals (CIs).

### Ethical considerations

This study was approved by the Ethics Committee of Shandong Provincial Third Hospital (Approval No: KYLL-2023068). Written informed consent was obtained from all participants. The study was conducted in accordance with the principles of the Declaration of Helsinki, and all personal data were de-identified to protect privacy.

## Results

### Study population and baseline characteristics

The study ultimately enrolled 258 patients with suspected UTI, of whom 152 (58.9%) were confirmed as infected by urine culture, and 106 (41.1%) served as the non-infected control group. Among the infected patients, 108 (71.1%) had Gram-negative bacterial infections, while 44 (28.9%) had Gram-positive bacterial infections. Normality testing confirmed non-normal distributions for all continuous variables (Shapiro-Wilk tests, all *p* < 0.05); therefore, continuous data are presented as median (interquartile range). As summarized in **Table 1**, no significant differences were observed between the infected and non-infected groups regarding age distribution (66.0 [50.0-73.0] years vs. 64.5 [51.2-76.0] years, *p* = 0.943) or gender (male: 50.0% vs. 52.8%, *p* = 0.748).

**Table 1:** Baseline characteristics of the study population.

Variable	Total	Infected	Non-infected	*P*-value
Demographic characteristics				
Age, years	65.5 (50.2-74.8)	66.0 (50.0-73.0)	64.5 (51.2-76.0)	0.943
Male gender	132 (51.2%)	76 (50.0%)	56 (52.8%)	0.748
Comorbidities				
Diabetes mellitus	81 (31.4%)	42 (27.6%)	39 (36.8%)	0.155
Hypertension	144 (55.8%)	78 (51.3%)	66 (62.3%)	0.106
Laboratory parameters				
PCT, ng/mL	0.08 (0.03-0.27)	0.06 (0.03-0.25)	0.13 (0.04-0.30)	0.030
CRP, mg/L	22.1 (7.4-64.5)	21.7 (7.0-60.9)	22.8 (8.0-73.3)	0.902
WBC count,/μL	241.2 (42.2-1177.9)	515.2 (70.1-1483.1)	65.9 (12.8-566.8)	<0.001
RBC count,/μL	32.3 (6.9-313.1)	31.0 (6.3-226.2)	35.0 (9.4-451.9)	0.403
BACT,/μL	3251.3 (202.4-29359.5)	16095.9 (3060.2-87294.8)	171.2 (15.0-1375.2)	<0.001
Urinary nitrite positive	91 (35.3%)	84 (55.3%)	7 (6.6%)	<0.001

Data are presented as median (interquartile range) for continuous variables and number (percentage) for categorical variables.

PCT, procalcitonin; CRP, C-reactive protein; WBC, white blood cell; RBC, red blood cell; BACT, bacterial count.

**Figure 1:**
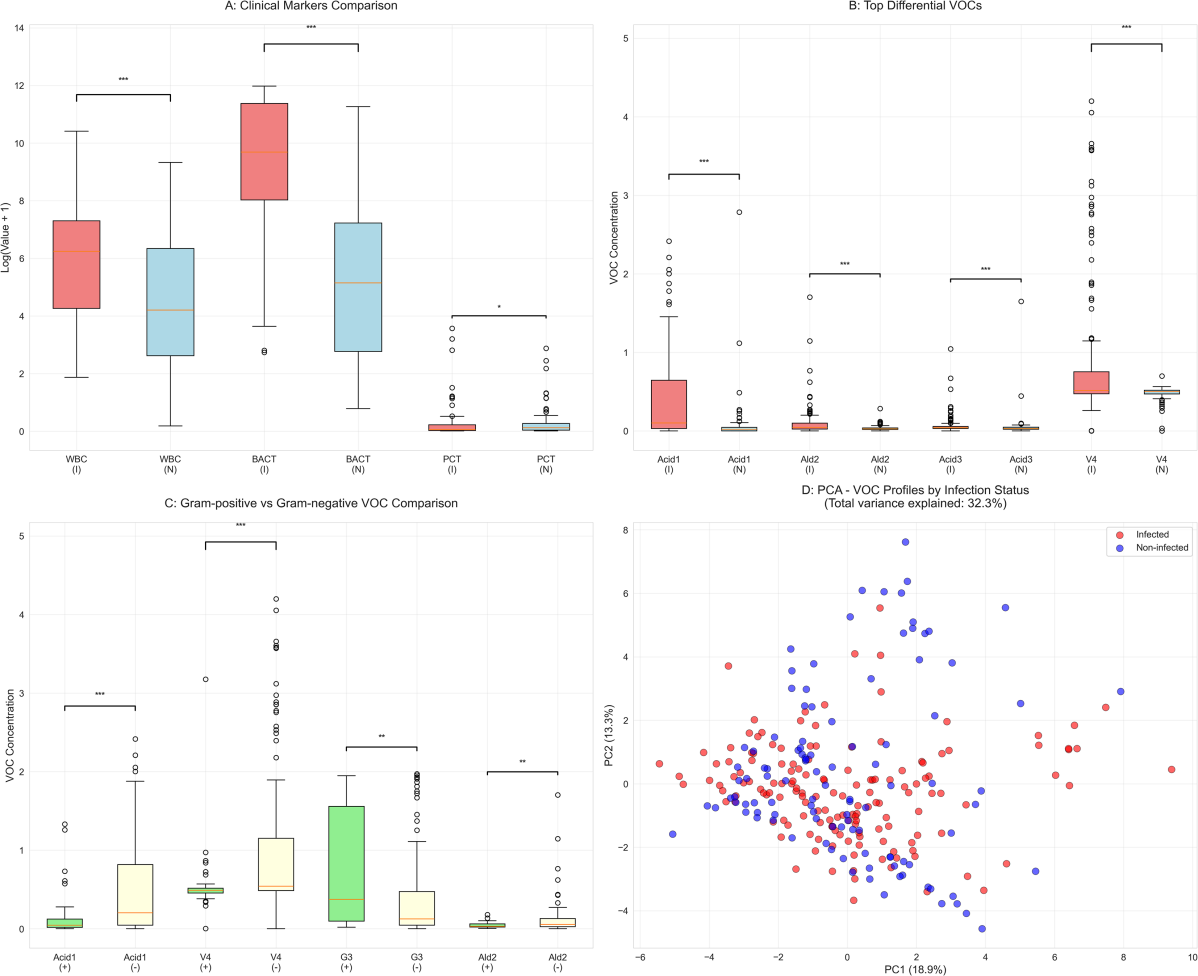
Group comparisons of clinical markers and urinary volatile organic compounds (VOCs). **(A)** Traditional clinical markers in infected (I) versus noninfected (N) groups. **(B)** Top 4 differential VOCs between groups. **(C)** Comparison of 4 VOCs between Gram-positive (+) and Gram-negative (-) bacterial infections. **(D)** Principal component analysis (PCA) of VOCs profiles colored by infection status. Abbreviations and symbols in panels **(B, C)**: Acid1, Acetic acid; Ald2, Benzaldehyde; Acid3: Propanoic acid; V4: furan; G3: Cyclohexanone-M; I, infected group; N, non-infected group; +, Gram-positive; –, Gram-negative. Statistical significance: *p < 0.05, **p < 0.01, ***p < 0.001.

In contrast, traditional infection markers were significantly elevated in the infected group (**Figure 1A**), including white blood cell (WBC) count (515.2 [70.1-1483.1]/μL vs. 65.9 [12.8-566.8]/μL, *p* < 0.001), bacterial count (BACT) (16095.9 [3060.2-87294.8]/μL vs. 171.2 [15.0-1375.2]/μL, *p* < 0.001), and procalcitonin (PCT) level (0.1 [0.0-0.2] ng/mL vs. 0.1 [0.0-0.3] ng/mL, *p* = 0.031). The positivity rate for urinary nitrite was also significantly higher in the infected group (55.3% vs. 6.6%, *p* < 0.001). The prevalence of comorbidities such as diabetes and hypertension did not differ significantly between the two groups.

### Identification and comparison of VOCs biomarkers

Systematic analysis of 33 urine VOCs identified 11 with differential expression between infection statuses (raw *p* < 0.05). After Bonferroni correction for multiple testing, three VOCs remained statistically significant. The detailed statistical results for all 33 VOCs are presented in **Supplementary Table S1**. As shown in **Figure 1B**, the most significantly altered VOCs included acetic acid (*p* = 1.22 × 10⁻¹², effect size η² = 0.106), benzaldehyde (*p* = 7.62 × 10⁻^7^, η² = 0.047), propanoic acid (*p* = 2.19 × 10⁻^5^, η² = 0.003), and furan (*p* = 9.29 × 10⁻^4^, η² = 0.083). In the Gram stain subgroup analysis, Gram-positive and Gram-negative bacterial infections also demonstrated distinct VOCs signatures. After multiple-testing correction, acetic acid (*p* = 0.0001) and furan (*p* = 0.0003) showed the most pronounced differences between these subgroups (**Figure 1C**). To visualize the high-dimensional VOCs data, principal component analysis (PCA) was performed. The first two principal components explained 18.94% and 13.33% of the total variance, respectively (**Figure 1D**). While a visual trend toward separation between infected and non-infected groups can be observed, there is substantial overlap, indicating that infection status cannot be reliably distinguished using these unsupervised components alone. This underscores the need for supervised machine learning methods for classification.

### Diagnostic model development and validation

**Figure 2:**
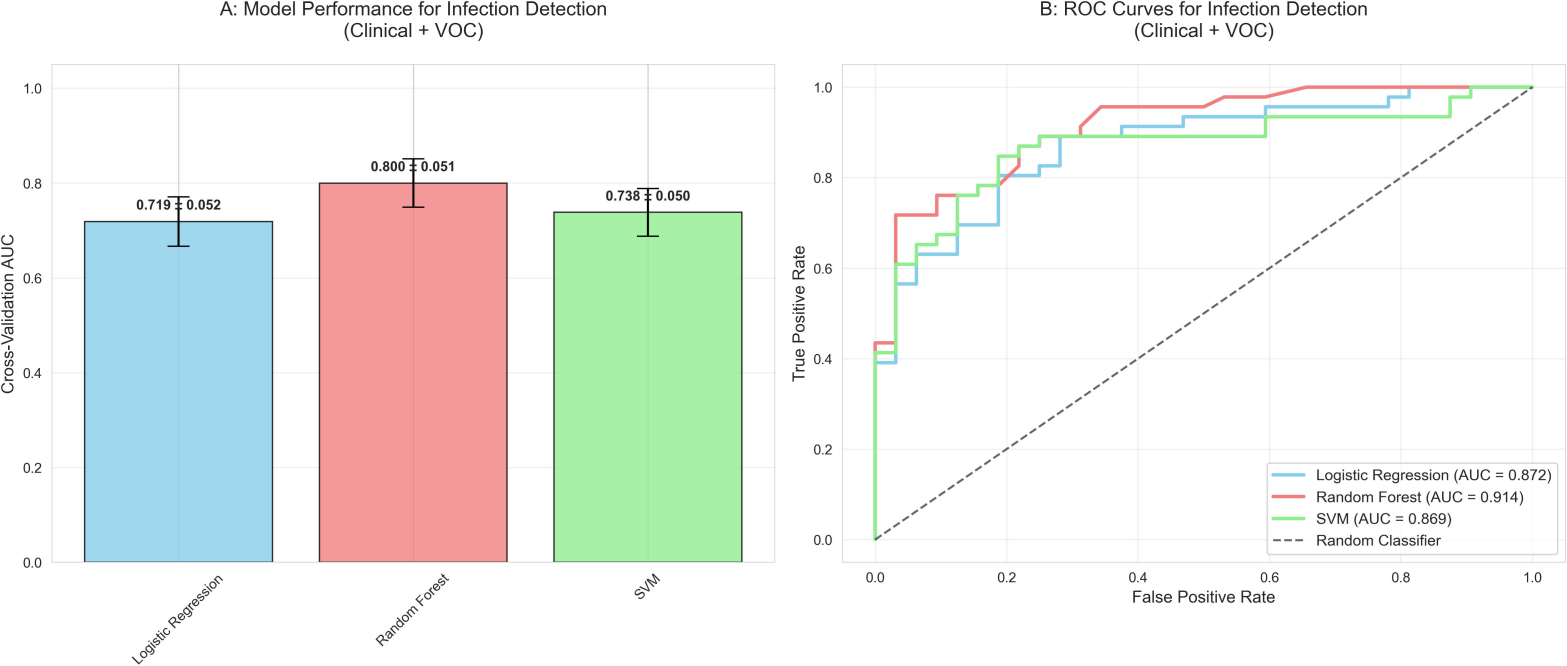
Diagnostic model performance for UTI detection using combined clinical and VOCs features. **(A)** Cross-validation AUC performance of three machine learning algorithms. **(B)** Receiver operating characteristic curves on the test set. LR, logistic regression; RF, Random Forest; SVM, Support Vector Machine.

We evaluated three machine learning algorithms using three feature sets, with comprehensive performance metrics reported in **Table 2**. The Random Forest algorithm demonstrated the most robust performance. For UTI diagnosis, the combined model (clinical + VOC) achieved a test set AUC of 0.914, with an accuracy of 82.1% (95% CI: 71.8-89.8%), sensitivity of 87.0%, specificity of 75.0%, and an F1-score of 0.851 (**Figure 2B**). This accuracy significantly exceeded the non-information rate (NIR) of 59.0% (binomial test, *p* < 0.001). Models using clinical-only or VOC-only attained test set AUCs of 0.831 and 0.850, respectively (**Figure 2A**).

**Table 2:** Diagnostic performance of machine learning models on the independent test set.

Feature set	Model	Test AUC	Accuracy	Sensitivity	Specificity	F1-score
Clinical-only	LR	0.833	0.756	0.652	0.906	0.759
	RF	0.831	0.744	0.739	0.750	0.773
	SVM	0.790	0.731	0.587	0.938	0.720
VOC-only	LR	0.798	0.718	0.674	0.781	0.738
	RF	0.850	0.744	0.804	0.656	0.787
	SVM	0.789	0.692	0.587	0.844	0.692
Clinical+VOC (Combined)	LR	0.872	0.795	0.804	0.781	0.822
	RF	0.914	0.821	0.870	0.750	0.851
	SVM	0.869	0.744	0.609	0.938	0.737

LR, Logistic Regression; RF, Random Forest; SVM, Support Vector Machine; AUC, area under the receiver operating.

Within the infected patient subgroup, VOCs-based models demonstrated varying performance in discriminating Gram-positive from Gram-negative infections. Logistic regression achieved the highest test set AUC of 0.800 with 76.1% accuracy (95% CI: 61.2-87.4%), while Random Forest and SVM models showed lower performance with AUCs of 0.709 and 0.718, respectively. These results provide preliminary evidence for the potential of VOCs to differentiate bacterial types, though performance remains moderate and requires further optimization.

### Comprehensive diagnostic performance evaluation

**Figure 3:**
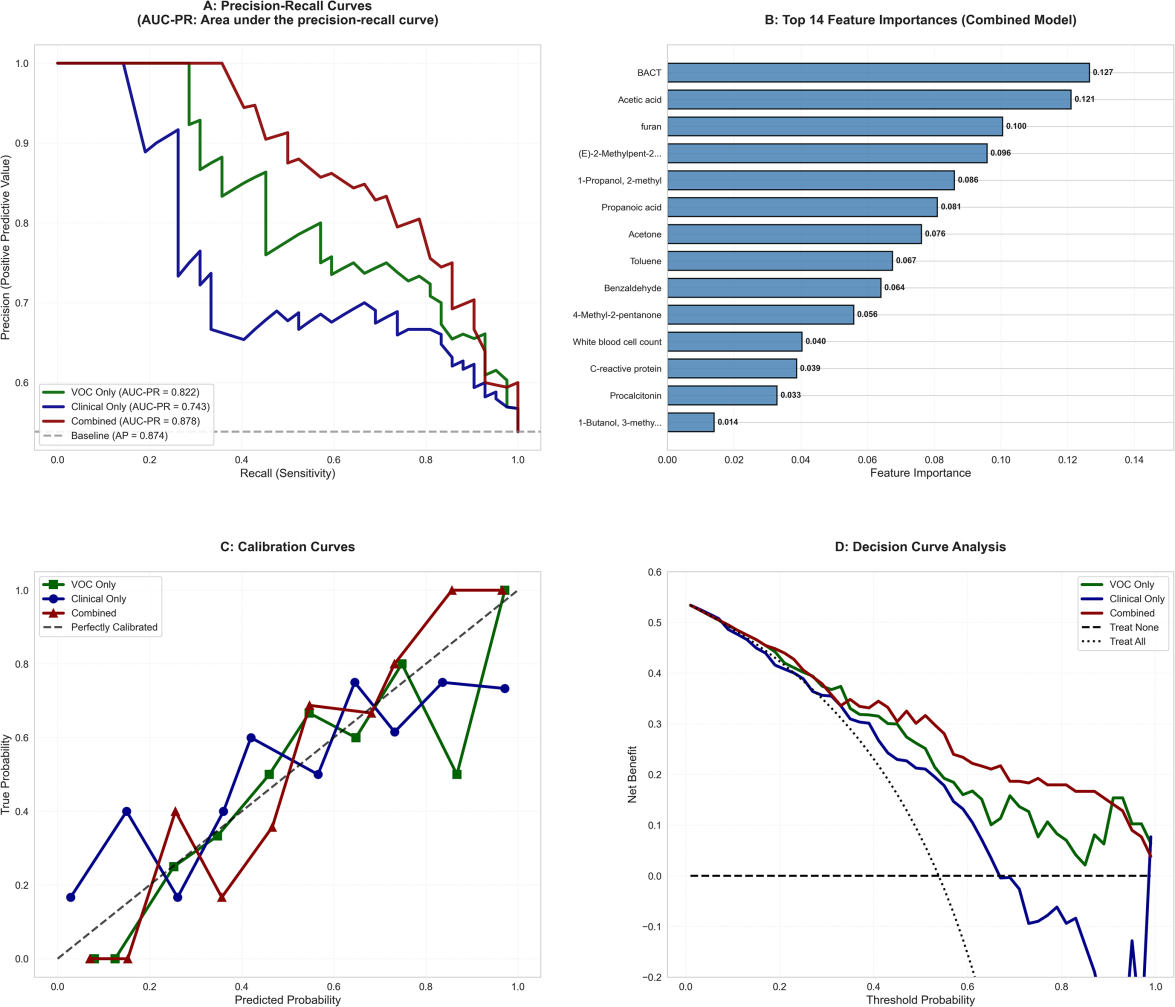
Comprehensive diagnostic performance evaluation of the optimal combined model. **(A)** Precision-recall curves showing the trade-off between sensitivity and positive predictive value (AUC-PR = area under the precision-recall curve). **(B)** Top 14 feature importances in the combined model. **(C)** Calibration curve comparing predicted probabilities with observed outcomes. **(D)** Decision curve analysis showing the clinical net benefit of using the model across different probability thresholds. BACT, bacterial count.

We conducted a comprehensive performance evaluation of the optimal combined model (Random Forest algorithm integrating clinical and VOCs features, test AUC = 0.914) In the precision-recall curve analysis, the combined model achieved an AUC-PR of 0.878, significantly outperforming the clinical-only (0.743) and VOC-only (0.822) models (**Figure 3A**). The calibration curve indicated excellent agreement between the predicted probabilities and the observed outcomes (**Figure 3C**). Decision curve analysis confirmed the clinical utility of the combined model, demonstrating a positive net benefit across a wide threshold probability range of 0.1 to 0.8 (**Figure 3D**). Feature importance analysis revealed 14 top predictors, 10 of which were VOCs biomarkers and 4 were clinical indicators, underscoring the substantial contribution of VOCs information to the model’s performance (**Figure 3B**).

### Multivariate analysis and independent predictors

**Figure 4:**
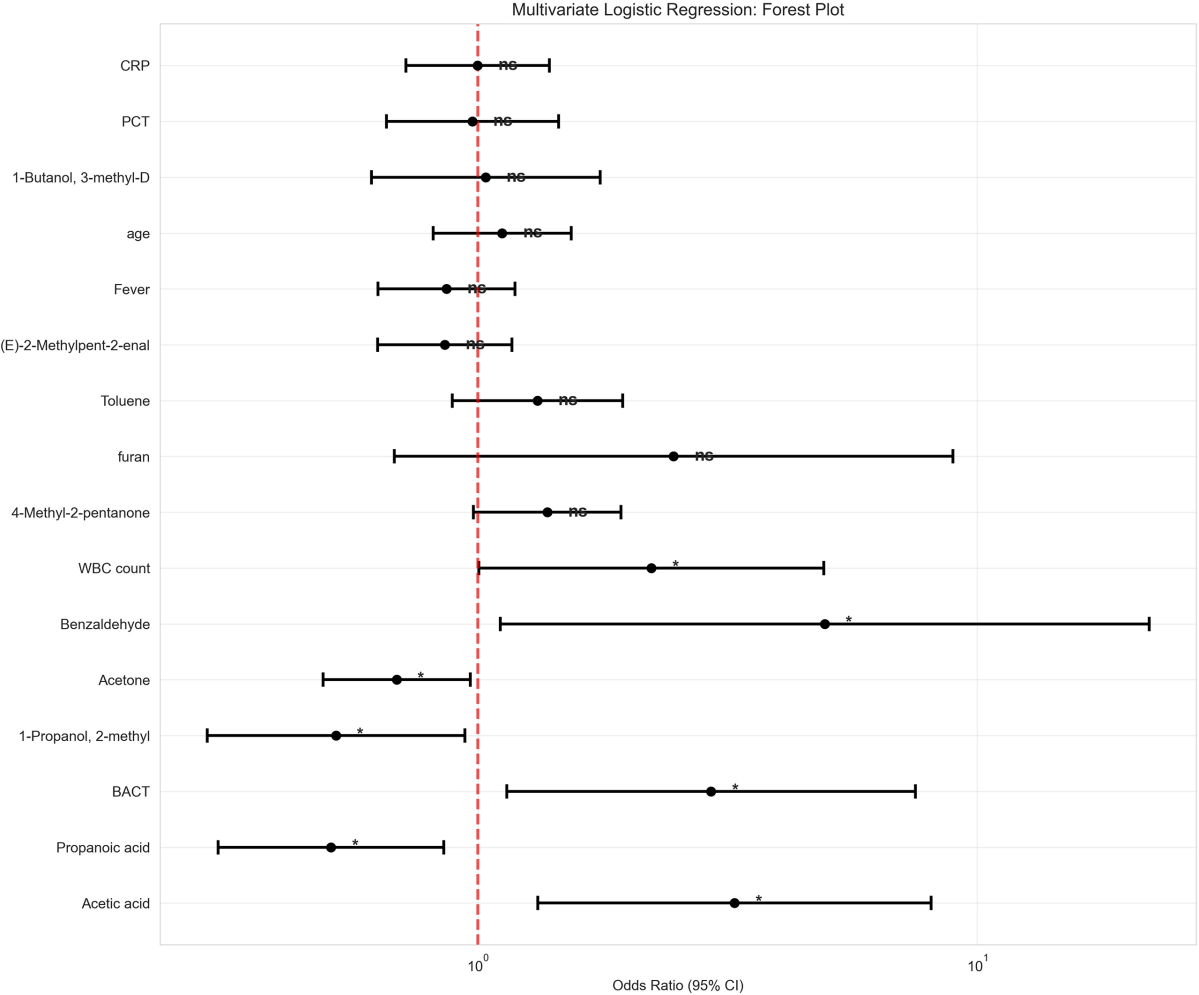
Forest plot of multivariate logistic regression analysis. Odds ratios and 95% confidence intervals for variables in the multivariate model. The reference line indicates no effect (OR = 1). BACT, bacterial count. Statistical significance: *p < 0.05.

Following LASSO-based feature selection, multivariate logistic regression analysis identified several independent predictors of UTI (**Table 3**; **Figure 4**). WBC count (OR = 2.23, 95% CI: 1.01-4.93, *p* = 0.048) and BACT (OR = 2.93, 95% CI: 1.14-7.53, *p* = 0.025) maintained independent predictive value as traditional infection markers. Among the VOCs biomarkers, acetic acid (OR = 3.27, 95% CI: 1.32–8.10, *p* = 0.010) and benzaldehyde (OR = 4.95, 95% CI: 1.11–22.11, *p* = 0.036) were identified as the strongest independent predictors, whereas acetone (OR = 0.69, 95% CI: 0.49–0.97, *p* = 0.031) and 2-methyl-1-propanol (OR = 0.52, 95% CI: 0.29–0.94, *p* = 0.031) exhibited protective effects.

**Table 3:** Multivariate logistic regression analysis for UTI prediction.

Variable	OR (95% CI)	*P*-value
Acetic acid	3.27 (1.32-8.10)	0.010
Propanoic acid	0.51 (0.30-0.86)	0.011
BACT	2.93 (1.14-7.53)	0.025
Acetone	0.69 (0.49-0.97)	0.031
2-Methyl-1-propanol	0.52 (0.29-0.94)	0.031
Benzaldehyde	4.95 (1.11-22.11)	0.036
White blood cell count	2.23 (1.01-4.93)	0.048

Odds ratios (OR) with 95% confidence intervals (CI) are presented. Variables with *p*<0.05 are considered statistically significant.

BACT, bacterial count.

### Pathogen-specific VOCs signature analysis

**Figure 5:**
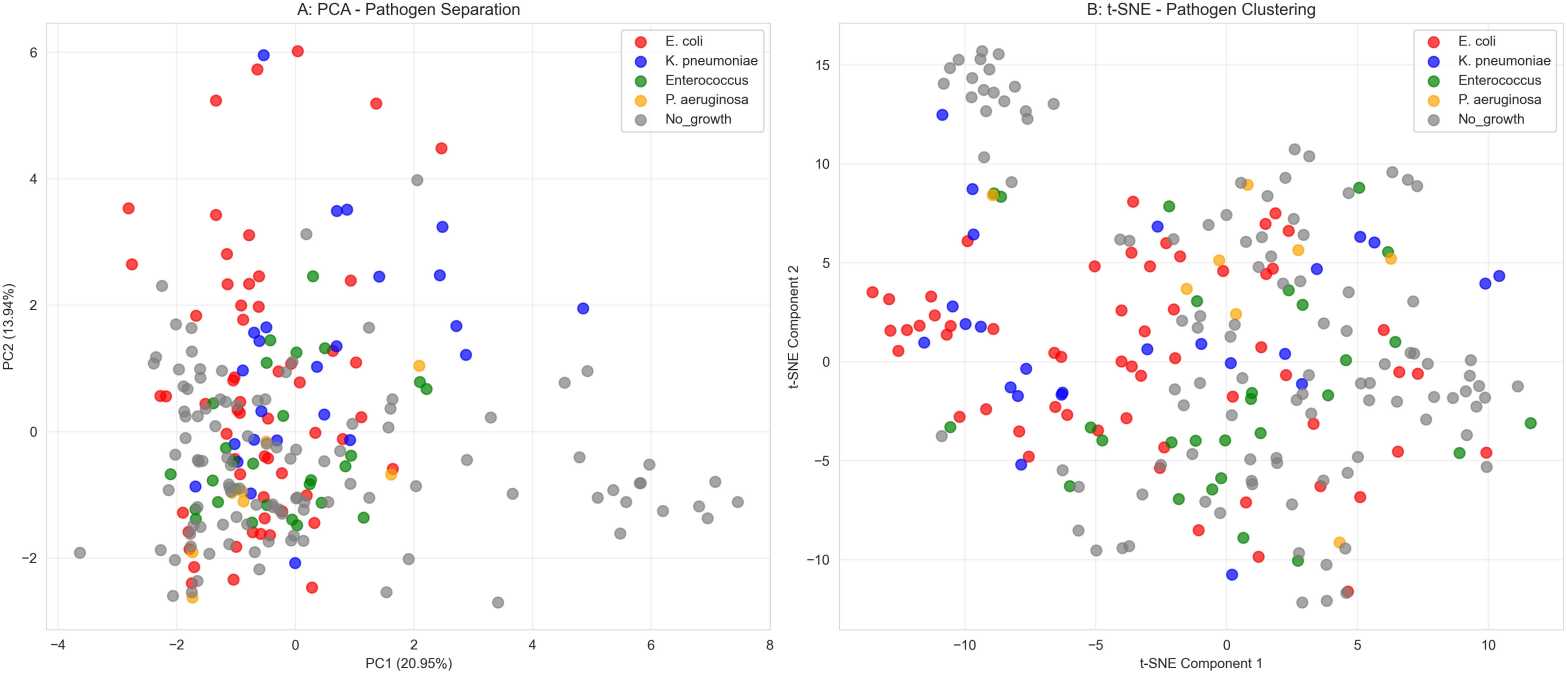
Pathogen-specific dimensionality reduction analysis. **(A)** Principal component analysis of VOCs profiles by pathogen type. **(B)** t-distributed stochastic neighbor embedding visualization. Each point represents an individual sample, colored by pathogen identity.

Among the 152 infected patients, we further analyzed the specific VOCs signatures of the predominant pathogens. The pathogen distribution was as follows: Escherichia coli (42.1%), Klebsiella pneumoniae (18.4%), Enterococcus spp. (12.5%), Pseudomonas aeruginosa (9.2%), Proteus mirabilis (6.6%), and other pathogens (11.2%). Both PCA and t-distributed stochastic neighbor embedding (t-SNE) visualizations showed some clustering of samples by pathogen type, though with considerable overlap between groups (**Figure 5**). Partial separation was observed between Gram-negative and Gram-positive bacteria in the reduced-dimensional space, consistent with the moderate discriminatory performance of the Gram classifier (AUC 0.70-0.80). These findings suggest that VOCs profiles may contain pathogen-specific information, though the degree of separation observed indicates that reliable pathogen discrimination would require further refinement.

### Biochemical pathway network analysis

By constructing a biochemical network of the significant VOCs (**Supplementary Figure S1**), we found that these compounds were primarily enriched in several key metabolic pathways: short-chain fatty acid metabolism (e.g., acetic acid, propanoic acid), aromatic amino acid metabolism (e.g., benzaldehyde, toluene), alcohol metabolism (various alcohols), and ketone body metabolism (e.g., acetone, 4-methyl-2-pentanone). Network analysis revealed significant co-expression relationships among these VOCs, suggesting they originate from interconnected microbial metabolic pathways and offering novel insights into the metabolic basis of UTIs.

## Discussion

The clinical management of UTIs faces the dual challenge of diagnostic delays and the escalating threat of antimicrobial resistance. Although the traditional urine culture remains the gold standard, its protracted turnaround time compels empiric antibiotic use, exacerbating global antibiotic misuse and resistance selection pressure (Wang and LaSala, 2021; Zhu et al., 2021). Our study prospectively integrated GC-IMS technology with advanced machine learning to systematically evaluate the diagnostic and typing value of urine VOCs in addressing this challenge. Our findings consistently demonstrate that the urine VOCs landscape, shaped by pathogen-host interactions, serves as a stable diagnostic resource. The primary novelty of this study lies in establishing an integrated, translatable diagnostic framework that extends beyond the identification of individual biomarkers. This framework is built upon three key elements: First, through prospective multimodal fusion, we demonstrated the significant incremental value of adding VOCs profiles to routine clinical parameters, achieving a combined model with high diagnostic performance (test AUC = 0.914). Second, we applied machine learning to develop a directly applicable predictive model, moving beyond associative analysis. Third, by constructing a biochemical pathway network, we linked VOCs signatures to core microbial metabolism, providing a mechanism-informed interpretation. Collectively, this framework represents a step toward a more precise approach to anti-infective therapy.

This study successfully identified a characteristic VOCs profile significantly enriched in the infected group, with acetic acid, benzaldehyde, and propanoic acid being particularly prominent. The appearance of these molecules is not random but is underpinned by robust microbial metabolic logic, elevating VOCs analysis beyond the limitation of traditional biomarkers that merely suggest “inflammation is present” to enabling the non-invasive monitoring of “which pathogen is metabolically active and how.” The strong independent predictive value of acetic acid (multivariate OR = 3.27) aligns closely with Clark’s classical description of mixed-acid fermentation in E. coli (Clark, 1989). As the predominant UTI pathogens, E. coli and other Enterobacteriaceae convert pyruvate-derived acetyl-CoA to acetate via the phosphate acetyltransferase (PTA) and acetate kinase (ACK) pathways in the microaerobic environment of the urinary tract, concurrently generating ATP. Thus, markedly elevated urinary acetate levels can be interpreted as direct ‘chemical evidence’ of vigorous fermentative metabolism by pathogens in the urinary environment. The enrichment of benzaldehyde likely stems from pathogen metabolism of aromatic amino acids. The classic work by TABAK et al (Tabak et al., 1964), using targeted screening and cultivation of phenol-adapted bacteria, systematically evaluated their degradation capacity for aromatic compounds and demonstrated their immediate and efficient oxidation of benzaldehyde. This provides *in vivo* support for such metabolic capabilities in the clinical setting. The enrichment of propanoic acid also has a solid foundation in microbial metabolism. Research indicates that the human colonic microbiota and various pathogenic bacteria can produce propanoic acid through multiple pathways, including the succinate and acrylate pathways (Louis and Flint, 2017). Notably, acetone and 2-methyl-1-propanol exhibited protective effects (OR < 1), suggesting their potential role as ‘protective’ metabolic markers. This finding can be interpreted from two angles: First, a decrease in these metabolites might signal suppressed metabolic activity of certain commensal microbes in the host gut that confer colonization resistance—these symbionts help maintain gut homeostasis through their metabolic products, and their dysfunction may indirectly increase UTI risk. Second, they might directly participate in host immune or metabolic regulation, and their decreased levels could reflect a dysregulation of host-protective metabolic pathways during infection. Although the concept of ‘protective metabolic markers’ is nascent in the UTI field, its core premise—that a healthy metabolic state of the host and its microbiota can produce molecules that resist pathogen colonization—is supported by broader research on microbial ecology and host interactions (Lawley and Walker, 2013; Zechner and Kienesberger, 2024). Our findings contribute novel evidence for this framework within the specific context of urinary tract infections.

A central contribution of this study lies in revealing the multiplicative effect of multimodal data fusion. While the clinical-only (AUC 0.831) and VOC-only (AUC 0.850) models each have their strengths, they possess inherent limitations: the former is susceptible to non-infectious inflammation, limiting specificity, while the latter can be influenced by ‘background noise’ from factors like diet and host basal metabolism (Capuano et al., 2025). When combined, the model leverages powerful and complementary information dimensions—clinical indicators sketch the macroscopic outline of the host’s systemic and local inflammatory response, while VOCs depict the microscopic metabolic fingerprint of pathogen community activity. This deep integration allows non-linear algorithms like Random Forest to capture more complex and disease-specific patterns, ultimately achieving a significant leap in diagnostic efficacy. This approach aligns with the emerging trend in complex infection diagnostics (e.g., sepsis, pneumonia) advocating for multimodal data integration (Frondelius et al., 2024; Shao et al., 2024). Decision curve analysis further affirmed, from a clinical utility perspective, that the combined model provides a positive net clinical benefit across a wide threshold probability range (0.1–0.8), solidifying its potential for integration into clinical decision support systems to guide more precise initial antibiotic selection.

Beyond establishing diagnostic value, our study explored the application of VOCs for the deeper clinical need of etiological differentiation. PCA and t-SNE visualizations indicated a partial separation between Gram-negative and Gram-positive bacterial infections in the VOCs feature space (**Figure 5**), although with considerable overlap. This finding is consistent with previous research. Tait et al. (2014) explicitly noted that VOCs profiles from Gram-positive bacteria (Staphylococcus aureus) differed fundamentally in diversity and composition from those of Gram-negative bacteria (E. coli, K. pneumoniae), with the former being more limited and lacking specific long-chain alcohols produced by the latter. Similarly, Frerichs et al. (2023) confirmed that fecal VOCs profiles had different predictive power and characteristic metabolites for Gram-negative versus Gram-positive late-onset sepsis in preterm infants. Although the current performance of the VOC-based Gram stain classifier (AUC 0.800) is only moderate and is by no means a substitute for standard susceptibility testing, it provides compelling proof-of-concept for a revolutionary ‘rapid etiological triage’. During the 24–48 hour window awaiting culture results, a rapid VOCs report available within hours suggesting “probable Gram-negative rod infection” could immediately guide clinicians toward targeted, narrow-spectrum antibiotics, significantly optimizing initial empiric therapy—a critical step toward precision anti-infective treatment.

To move beyond a mere listing of discrete molecules, we integrated the identified significant VOCs into a coherent, biologically meaningful ‘metabolic story’ by constructing a biochemical pathway network. These molecules were significantly enriched in a few core pathways: short-chain fatty acid metabolism, aromatic amino acid metabolism, and ketone/alcohol metabolism. This networked, pathway-oriented analysis has dual significance: First, it substantially enhances the biological interpretability of our results, directly linking observed VOCs changes to known microbial biochemical pathways, thereby elevating our findings from purely ‘data-driven’ to possessing a ‘mechanism-informed’ dimension (Sevin et al., 2015). Second, it suggests that future diagnostic models could potentially move beyond reliance on the abundance of single, potentially volatile molecules to focus on the more robust assessment of metabolic pathway functionality, which may significantly improve model stability and generalizability across individual variations (e.g., diet, medication, comorbidities) and complex clinical settings (Matthews et al., 2016).

We openly acknowledge the limitations of our study. First, the diagnostic performance of our model was benchmarked against conventional urine culture, the widely accepted clinical gold standard. It is important to note that this reference standard itself is not infallible. Factors such as prior antibiotic use, the presence of fastidious microorganisms, or asymptomatic bacteriuria could lead to misclassification of some patients. For instance, true infections with culture-negative results would have been assigned to the control group, an unavoidable bias that may lead to an underestimation of the model’s true discriminatory power. Second, as a single-center investigation, its participant population and pathogen spectrum may reflect regional characteristics. The generalizability of our conclusions requires validation in multi-center, large-scale, prospective external cohorts encompassing broader geographical regions, diverse populations, and healthcare settings—a necessary step for translational diagnostic research (Yang et al., 2022). Third, while GC-IMS offers significant advantages in sensitivity and speed, its ability to precisely resolve isomeric compounds remains challenging, and standardized protocols are currently lacking. Future complementary analysis using gas chromatography-mass spectrometry (GC-MS) would provide higher specificity for the absolute quantification and precise identification of key biomarkers, facilitating the development of diagnostic models transferable across platforms (Capitain and Weller, 2021; Epping and Koch, 2023). For instance, the thermal desorption GC-MS approach used by Ahmed et al. (2023) offers a rigorous methodological reference for future precise identification and clinical translation of our biomarkers. Fourth, some laboratory parameters had missing data (PCT: 40.3%, CRP: 40.3%), which reflects real-world clinical practice where these tests are selectively ordered based on disease severity. However, our primary analyses focused on VOC biomarkers that had complete data for all participants. Fifth, our model did not account for all potential confounding sources of urinary VOCs, such as host metabolic states (e.g., diabetic ketosis), diet, or medications. Although we recorded and adjusted for diabetes status statistically, the potential influence of these factors may affect specificity. Sixth, the study did not clinically stratify patients into upper versus lower urinary tract infections. Differences in pathogen burden and host response could influence VOCs profiles. Seventh, while we focused on microbial origins, the identified VOCs may also originate from or be modulated by host metabolism. Disentangling this contribution remains a challenge. Eighth, our combined model incorporated established clinical markers (e.g., nitrite) alongside novel VOCs. While this could theoretically introduce incorporation bias, the significant performance gain with VOCs confirms their substantial incremental value.

Finally, although we successfully constructed high-accuracy VOC-based models for UTI diagnosis and pathogen typing, our attempt to explore their potential for discriminating more complex phenotypes—such as extended-spectrum β-lactamase (ESBL)-producing isolates—yielded suboptimal results (AUC < 0.6). This outcome partially aligns with the findings of Smart et al (Dospinescu et al., 2020), who noted that distinguishing resistance requires higher-resolution metabolic profiles and that resistance-related metabolic alterations might be masked by the inherent VOC differences between species. Future studies will require larger cohorts focused on specific pathogens (e.g., E. coli only) and potentially the integration of more sensitive targeted metabolomics or multi-omics data to successfully capture the volatile ‘fingerprint’ specific to antimicrobial resistance (Lammers et al., 2022).

## Conclusion

In summary, through a methodologically rigorous prospective cohort study with appropriate non-parametric statistical analyses, we have established urine VOCs analysis via GC-IMS as a robust, non-invasive, rapid, and information-rich diagnostic tool for UTIs. The developed and validated clinical-VOCs combined model not only demonstrates excellent discriminatory capability but, more importantly, the pathogen-specific information and metabolic pathway context embedded within the VOCs signatures open new avenues for understanding infection mechanisms and advancing towards rapid pathogen differentiation. With further technological standardization and large-scale validation, diagnostic strategies incorporating VOCs analysis hold the potential to reshape the clinical pathway for UTI, ushering antimicrobial stewardship into a more precise, timely, and sustainable era.

## Data Availability

The raw data supporting the conclusions of this article will be made available by the authors, without undue reservation.
