# Supplementary material for: Urine volatile organic compounds profiling via GC-IMS combined with machine learning: a powerful diagnostic and pathogen differentiation tool for urinary tract infections
Source: Front Cell Infect Microbiol. 2026 Feb 11;16:1745468. doi: 10.3389/fcimb.2026.1745468 (PMC12932616; doi:10.3389/fcimb.2026.1745468)
Supplement: Supplementary file 1 [file DataSheet1.docx]

**Table S1. Complete statistical analysis of 33 urinary VOCs between UTI-infected and non-infected groups**

| VOC | *p*-value | η² | U statistic | *p* adj (Bonferroni) |
| --- | --- | --- | --- | --- |
| Acetic acid | <0.001 | 0.106 | 50.45 | <0.001 |
| Benzaldehyde | <0.001 | 0.047 | 24.45 | <0.001 |
| Propanoic acid | <0.001 | 0.003 | 18.02 | 0.001 |
| Furan | 0.001 | 0.083 | 10.96 | 0.031 |
| Acetone | 0.008 | 0.021 | 6.99 | 0.27 |
| 4-Methyl-2-pentanone | 0.013 | 0.006 | 6.24 | 0.413 |
| 2-Methyl-1-propanol | 0.017 | 0.053 | 5.7 | 0.561 |
| 3-Methyl-1-butanol (D) | 0.02 | 0.031 | 5.43 | 0.654 |
| (E)-2-Methylpent-2-enal | 0.025 | 0.028 | 5.02 | 0.826 |
| Toluene | 0.027 | 0.032 | 4.89 | 0.892 |
| 1-Propanol (M) | 0.043 | 0.007 | 4.11 | 1 |
| Hexanal (D) | 0.069 | 0.002 | 3.32 | 1 |
| Cyclohexanone (M) | 0.092 | 0.001 | 2.83 | 1 |
| Propanal | 0.136 | 0.01 | 2.22 | 1 |
| Dimethyl sulfide | 0.153 | 0.002 | 2.04 | 1 |
| 1-Propanol (D) | 0.203 | 0.007 | 1.62 | 1 |
| 1-Nonanal | 0.217 | 0.003 | 1.53 | 1 |
| Methanol | 0.28 | 0.001 | 1.17 | 1 |
| 3-Methyl-1-butanol (M) | 0.371 | 0.025 | 0.8 | 1 |
| Ethanol | 0.386 | 0.005 | 0.75 | 1 |
| 2-Methyl-2-propanol | 0.474 | <0.001 | 0.51 | 1 |
| 3-Heptanone | 0.494 | 0.004 | 0.47 | 1 |
| 2-Pentanone | 0.501 | 0.001 | 0.45 | 1 |
| 3-Hexanone (M) | 0.52 | 0.001 | 0.41 | 1 |
| 2-Butanone | 0.524 | 0.002 | 0.41 | 1 |
| 2-Heptanone (M) | 0.625 | <0.001 | 0.24 | 1 |
| 2-Heptanone (D) | 0.655 | 0.015 | 0.2 | 1 |
| Hexanal (M) | 0.664 | <0.001 | 0.19 | 1 |
| 2-Ethyl hexanol | 0.727 | <0.001 | 0.12 | 1 |
| (E)-3-Hexen-1-ol | 0.778 | 0.005 | 0.08 | 1 |
| Cyclohexanone (D) | 0.888 | <0.001 | 0.02 | 1 |
| 3-Hexanone (D) | 0.913 | 0.001 | 0.01 | 1 |
| 4-Heptanone | 0.957 | <0.001 | 0 | 1 |

Statistical analysis was performed using the Mann-Whitney U test. η² denotes effect size. The *p*_adjusted values were calculated using the Bonferroni correction for 33 multiple comparisons; values greater than 1.000 are presented as 1.000. (M) and (D) denote monomer and dimer peaks detected by GC-IMS, respectively.


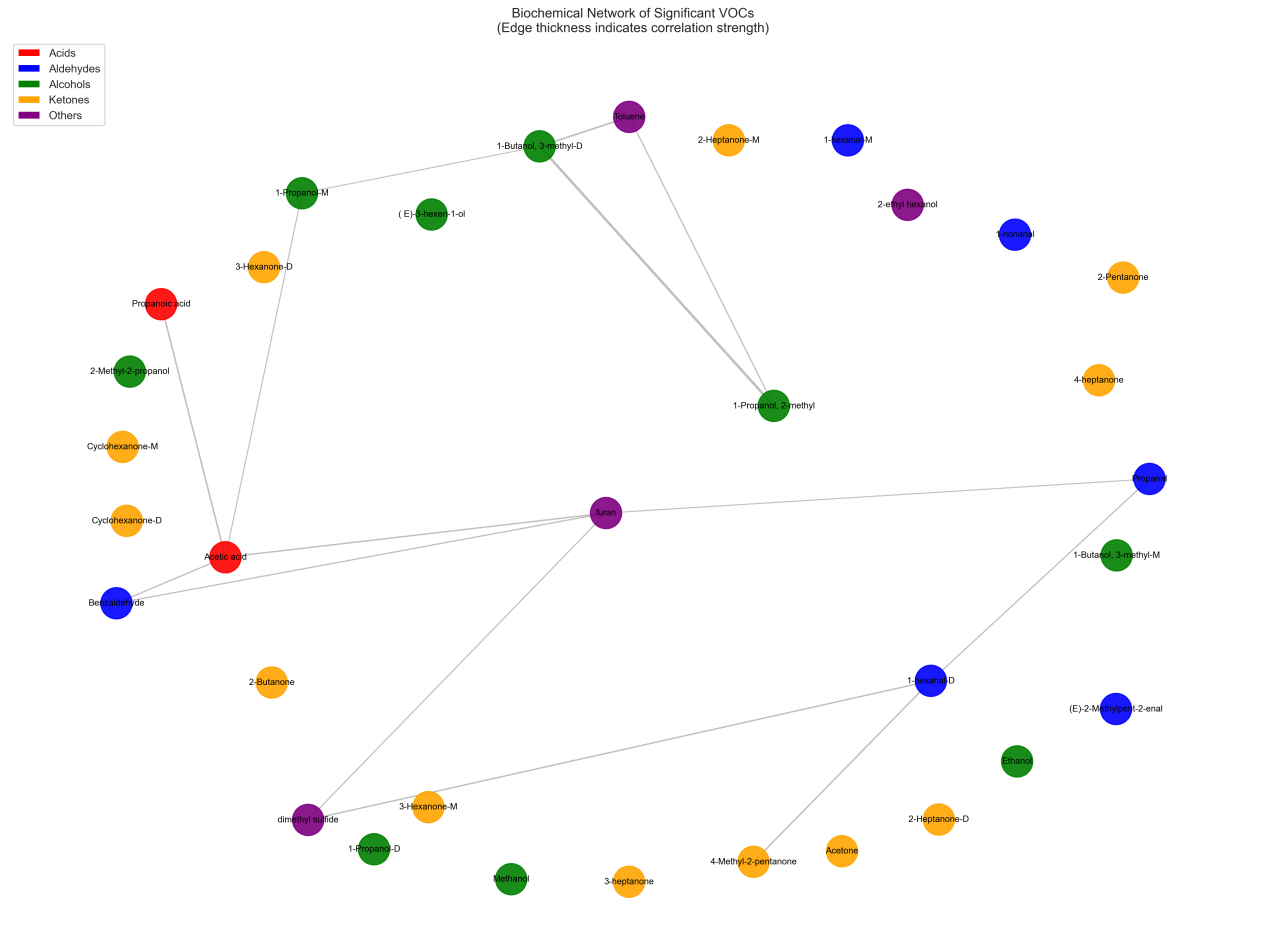


**Figure S1. Co-expression network of differential volatile organic compounds (VOCs) in UTI.**

Nodes represent VOCs significantly associated with infection (Bonferroni-adjusted *p* < 0.05), colored by chemical class. Edges connect VOCs with significant pairwise correlations (Pearson |r| > 0.3, *p* < 0.05).
